# A global perspective of the changing epidemiology of invasive fungal disease and real-world experience with the use of isavuconazole

**DOI:** 10.1093/mmy/myae083

**Published:** 2024-08-13

**Authors:** George R Thompson, Sharon C-A Chen, Wadha Ahmed Alfouzan, Koichi Izumikawa, Arnaldo L Colombo, Johan Maertens

**Affiliations:** Department of Internal Medicine, Division of Infectious Disease, UC Davis Medical Center, Sacramento, California, USA; Department of Medical Microbiology and Immunology, University of California, Davis, California, USA; Centre for Infectious Diseases and Microbiology Laboratory Services, New South Wales Health Pathology, and the Department of Infectious Diseases, Westmead Hospital, School of Medicine, University of Sydney, Sydney, New South Wales, Australia; Department of Laboratories, Farwaniya Hospital, Farwaniya, Kuwait; Department of Microbiology, College of Medicine, Kuwait University, Kuwait City, Kuwait; Department of Infectious Diseases, Nagasaki University Graduate School of Biomedical Sciences, Nagasaki, Japan; Division of Infectious Diseases, Escola Paulista de Medicina, Universidade Federal de São Paulo, São Paulo, Brazil; Antimicrobial Resistance Institute of São Paulo, São Paulo, Brazil; Department of Microbiology, Immunology and Transplantation, KU Leuven and Department of Hematology, University Hospitals Leuven, Leuven, Belgium

**Keywords:** Fungal epidemiology, healthcare resource utilization, invasive fungal disease, isavuconazonium sulfate, antifungal therapy, real-world

## Abstract

Global epidemiological data show that the incidence of invasive fungal disease (IFD) has increased in recent decades, with the rising frequency of infections caused by *Aspergillus* and Mucorales order species. The number and variety of patients at risk of IFD has also expanded, owing in part to advances in the treatment of hematologic malignancies and other serious diseases, including hematopoietic stem cell transplantation (HCT) and other therapies causing immune suppression. Isavuconazonium sulfate (active moiety: isavuconazole) is an advanced-generation triazole antifungal approved for the treatment of invasive aspergillosis and mucormycosis that has demonstrated activity against a variety of yeasts, moulds, and dimorphic fungi. While real-world clinical experience with isavuconazole is sparse in some geographic regions, it has been shown to be effective and well tolerated in diverse patient populations, including those with multiple comorbidities who may have failed to respond to prior triazole antifungal therapy. Isavuconazole may be suitable for patients with IFD receiving concurrent QTc-prolonging therapy, as well as those on venetoclax or ruxolitinib. Data from clinical trials are not available to support the use of isavuconazole prophylactically for the prevention of IFD or for the treatment of endemic IFD, such as those caused by *Histoplasma* spp., but real-world evidence from case studies suggests that it has clinical utility in these settings. Isavuconazole is an option for patients at risk of IFD, particularly when the use of alternative antifungal therapies is not possible because of toxicities, pharmacokinetics, or drug interactions.

## Introduction

The incidence and epidemiology of invasive fungal diseases (IFDs) are continuing to evolve. A worldwide problem, IFDs caused by many pathogens can be difficult to treat and cure because of reduced susceptibility or resistance to current antifungal agents and are associated with high mortality rates: An estimated 6.5 million patients are affected by IFDs each year,^1^ leading to approximately 2.5 million deaths annually.^1^ However, accurate figures for IFD are difficult to obtain owing in part to the complexities of diagnosis, leading the World Health Organization (WHO) to conclude there is an underestimation of the global burden of these infections.^2^

Advances in medicine have led to increased survival among patients with serious illnesses, but consequently, there has been a rise in the number of individuals with impaired immune function or those with invasive medical interventions at risk from opportunistic pathogenic fungi.^3,4^ In patients with hematological malignancies, especially those undergoing allogeneic hematopoietic stem cell transplantation (HCT), IFD is a common cause of morbidity and mortality.^5^

Isavuconazole is a US food and drug administration (FDA)-approved advanced-generation triazole antifungal indicated in adult patients for the management of invasive aspergillosis (IA) and invasive mucormycosis (IM) since 2015.^6^ Most recently, isavuconazole became the first azole antifungal therapy approved by the FDA for pediatric patients in 2023, and it remains the only therapy available in children affected by potentially life-threatening IA and IM.^6^ It has also been approved by the european medicines agency (EMA) for use in patients over 1 year of age in 2024.^7^

As the epidemiology of IFDs is evolving and with newer patient risk groups identified,^3–5^ this article reviews the existing evidence on the epidemiology and risk factors for IFD, discusses the effectiveness and safety of isavuconazole for the treatment of IA and IM, and examines its potential to prevent IFD in specific patient populations based on clinical data from real-world studies.

## Incidence and epidemiology of invasive fungal infections

**Figure 1.**
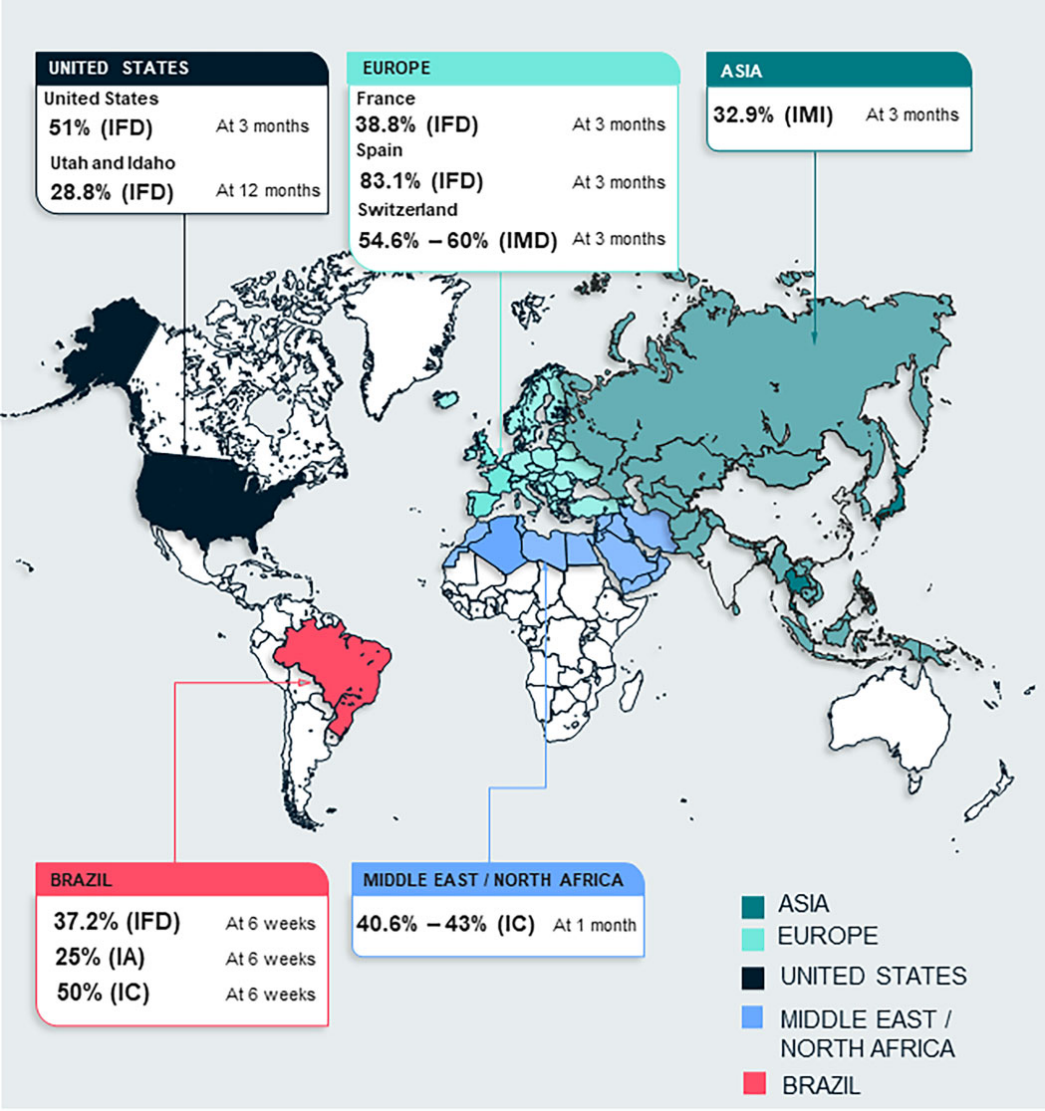
Regional mortality rates^a^ attributed to invasive fungal disease at 3 months (unless otherwise specified).^9,12,14,15,20–22,26^ ^a^The definition of mortality varied across the studies; please refer to Table 1 for details. IA, invasive aspergillosis; IC, invasive candidiasis; IFD, invasive fungal infections; IMD, invasive mould disease; IMI, invasive mould infection.

**Figure 2.**
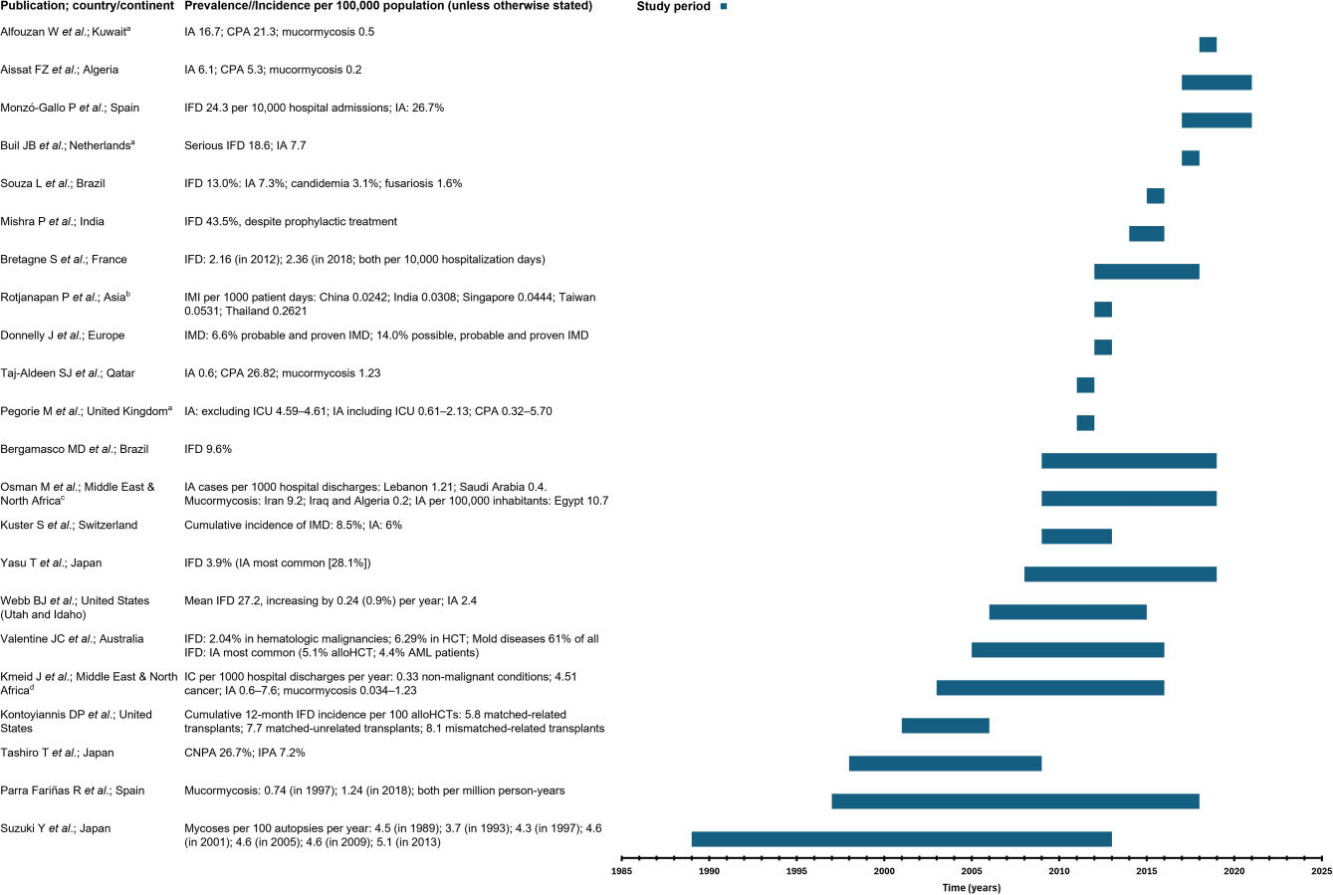
Incidence or prevalence of invasive fungal disease across different geographies over time.^8–28,127^ ^a^Study covered various periods, with results extrapolated to the total population of the respective country in the year shown in the figure; ^b^Countries included: China, India, Singapore, Taiwan, and Thailand; ^c^Countries included: Egypt, Lebanon, Saudi Arabia, Iran, Iraq, and Algeria; ^d^Range of studies included. alloHCT, allogeneic hematopoietic stem cell transplantation; AML, acute myeloid leukemia; CNPA, chronic necrotizing pulmonary aspergillosis; CPA, chronic pulmonary aspergillosis; HCT, hematopoietic stem cell transplantation; IA, invasive aspergillosis; IC, invasive candidiasis; ICU, intensive care unit; IFD, invasive fungal disease; IMD, invasive mould disease; IMI, invasive mould infection; IPA, invasive pulmonary aspergillosis.

The incidence of IFD in adults varies by geographical region and may reflect differences in clinical practices and patient populations (Table 1, Fig. 1). For Europe as a whole, recent incidence data are scarce; in 2014, a European-wide period prevalence study reported varying incidence of IA among patients with acute myeloid leukemia (AML) or myelodysplastic syndrome (MDS) according to the timing of therapy: 9% with induction therapy, 4% with consolidation therapy, and 4.7% in recipients of HCT.^8^ Incidence and prevalence data available for individual countries and regions are illustrated in Figure 2; these data include European countries, where a rise in incidence of IFD and of IA and mucormycosis in particular has been reported.^8–14^

**Table 1.** Overview of the incidence of invasive fungal disease by geographical region.

Region/country	Incidence/prevalence	Time period	Patients/episodes/cases (*N*)	Mortality	Study type	Comments
Europe (PIMDA)^8^	IMD across Europe: 6.6% probable and proven IMD; 14.0% possible, probable, and proven IMD	2012 (June 6 to December 5)	Patients: 1205	NR	Prospective IMD audit	Patients with AML or MDS, or conditioning therapy to prepare for an alloHCT
France^9^	IFD incidence: 2.16 per 10 000 hospitalization days in 2012; 2.36 per 10 000 hospitalization days in 2018	2012–2018	Patients: 10 154. IFD (episodes): 10 886	Overall (at 3 months): 38.8%	Active surveillance	Most (95.9%) patients were adults; the most common underlying conditions were malignancies (43.2%)
United Kingdom^10^	IA prevalence (all risk groups, except ICU; per 100 000 population): 4.59–4.61. IA prevalence (all risk groups, including ICU; per 100 000 population): 0.61–2.13. CPA prevalence (all risk groups; per 100 000 population): 0.32–5.70	Various	IA (cases; all risk groups except ICU): 2901–2912. IA (cases; all risk groups including ICU): 387–1345. CPA (cases; all risk groups): 204–3600	NR	Epidemiologic; estimates based on published incidence rates in the UK, extrapolated to 2011 population size	Risk groups included ICU patients, patients with autoimmune disease, liver failure; other conditions treated with CS; and solid tumors other than lung tumors
Netherlands^11^	Serious IFD incidence (per 100 000 population): 18.6. IA incidence (per 100 000 population): 7.7	2017	IFD (per year): 3185. IA (per year): 1283	NR	Epidemiologic; estimates based on published incidence rates in the Netherlands, extrapolated to 2017 population size	–
Spain^12^	IFD incidence (cases per 10 000 hospital admissions): 24.3. IA incidence: 26.7% of cases and was the most common IFD (36.7%) among patients with hematologic malignancies	2017–2021	IFD (episodes among 150 740 hospital admissions): 367	IFD-attributable mortality (at 12 weeks): 83.1% (*n* = 118)	Single center, retrospective, observational	700-bed university hospital serving 500 000 inhabitants; reference center for hematologic and solid malignancies, complex and ICU cases, and alloHCT recipients
Spain^13^	Mucormycosis incidence (per million person-years): 0.74 in 1997; 1.24 in 2018	1997–2018	Mucormycosis (cases): 29 in 1997; 58 in 2018	31.3%	Retrospective, longitudinal, descriptive study based on NHS in Spain	Included all hospital admissions with principal and/or secondary diagnosis of mucormycosis
Switzerland^14^	IMD cumulative incidence: 8.5%. IA cumulative incidence: 6%. Non-*Aspergillus* IMD cumulative incidence: 2.5%	2009–2013	IFD (cases among 479 alloHCT recipients): 41. IMD (cases, including 20 IA cases): 31	IMD (probable/proven at 12 weeks): 58%. IA (probable/proven at 12 weeks): 60%. Non-*Aspergillus* IMD (probable/proven at 12 weeks): 54.6%	Retrospective cohort study of alloHCT recipients included in the European Group for Bone Marrow Transplantation and Swiss Transplant Cohort Study	Cases had probable or proven infection
Middle East and North Africa^15^	IC incidence (per 1000 hospital discharges per year): 0.33 in patients with non-malignant conditions to 4.51 in patients with cancer. IA incidence (per 100 000 inhabitants): 0.6–7.6. Mucormycosis incidence (per 100 000 inhabitants per year): 0.034–1.23	Various (2003–2016)	NR	IC cases (crude mortality; at 30 days): 40.6%–43%. Non-*albicans Candida* (at 12 months): 57.8%–81.9%	Literature review of published studies	Data sourced from multiple published studies; sparse data for IA and mucormycosis
Kuwait^16^	IA incidence (per 100 000 population per year): 16.7. CPA incidence (per 100 000 population per year): 21.3. Mucormycosis incidence (per 100 000 population per year): 0.5	2018	IA (cases): 704. CPA (cases): 995. Mucormycosis (cases): 23	NR	Systematic literature search and data sourced from a reference mycology laboratory	Incidence estimates based on 2018 population data
Qatar^17^	IA incidence (per 100 000 population): 0.6. CPA incidence (per 100 000 population): 26.82. Mucormycosis incidence (per 100 000 population): 1.23	2011	IA (cases): 11. CPA (cases): 176. Mucormycosis (cases): 23	NR	Microbiology database and clinical characteristics for in/outpatients of private hospitals and clinics	–
Middle East and North Africa^18^	IA incidence: Egypt (per 100 000 inhabitants), 10.7; Lebanon (cases per 1000 hospital discharges), 1.21; Saudi Arabia (cases per 1000 hospital discharges), 0.4. Mucormycosis incidence: Iran (per 100 000 population), 9.2; Iraq and Algeria (per 100 000 population), 0.2	2009–2019	NR	NR	Literature search	–
Algeria^19^	IA incidence (per 100 000 population): 6.1. CPA prevalence (per 100 000 population): 5.3. Mucormycosis incidence (per 100 000 population): 0.2	2017–2021	IA (cases): 2639. CPA (cases): 10 788. Mucormycosis (cases): 87	NR	Review of published literature and results from relevant conferences	–
United States^20^	IFD 12-month cumulative incidence (per 100 alloHCTs): 5.8 in matched-related transplants; 7.7 in matched-unrelated transplants; 8.1 in mismatched-related transplants	2001–2006	IFD (in 639 HCT recipients): 718	All-cause mortality (at 3 months): 51%	Prospective surveillance data from transplant centers in the TRANSNET database	–
United States (Utah and Idaho)^21^	IFD mean incidence (cases per 100 000 patients per year): 27.2, increasing year-on-year by 0.24 cases per 100 000 patients (0.9%) per year. IA mean incidence (cases per 100 000 patients per year): 2.4	2006–2015	IFD (episodes in 3154 patients): 3374	IFD (all-cause 1-year mortality): 28.8%. IA (all-cause 1-year mortality): 48.8%	Retrospective review of a healthcare network database	–
Brazil^22^	IFD incidence: 9.6%	2009–2019	IFD (cases in 980 patients with hematologic malignancies and HCT recipients): 94	Overall (all-cause 6-week mortality): 37.2%. Aspergillosis (all-cause 6-week mortality): 25%. Fusariosis (all-cause 6-week mortality): 50%. Candidiasis (all-cause 6-week mortality): 50.0%	Retrospective cohort study from a single tertiary care center	–
Brazil^23^	IFD incidence (cases per year): 13.0%. IA incidence (cases per year): 7.3%. Candidemia incidence (cases per year): 3.1%. Fusariosis incidence (cases per year): 1.6%	2015–2016	IFD (proven/probable cases among 192 patients with hematologic malignancies): 25. IA (proven/probable cases among 192 patients with hematologic malignancies): 14. Candidemia (proven/probable cases among 192 patients with hematologic malignancies): 6. Fusariosis (proven/probable cases among 192 patients with hematologic malignancies): 3	NR	Prospective, multicenter cohort study	IFDs included cases of proven and probable infection
Japan^24^	Mycoses prevalence (cases per 100 autopsies per year): 4.5 in 1989; 3.7 in 1993; 4.3 in 1997; 4.6 in 2001; 4.6 in 2005; 4.6 in 2009; 5.1 in 2013	1989–2013	Visceral mycoses (cases in 164 764 autopsied cases): 7194	NR	Review of autopsy data	–
Japan^127^	IFD incidence: 3.9%. IA incidence (most common IFD): 28.1%	2008–2019	IFD (in 3484 patients with CLL): 135. IA (in 135 patients with CLL): 38	NR	Retrospective cohort study of nationwide database	–
Japan^25^	CNPA: 26.6%. IPA: 7.2%	1998–2009	Clinical isolates of *Aspergillus spp*. (among 139 patients): 165	IA (in-hospital mortality): 80%. CPA (in-hospital mortality): 16%	Retrospective study of hospital records	–
China, India, Singapore, Taiwan, Thailand^26^	IMI incidence (per 1000 patient days): China, 0.0242; India, 0.0308; Singapore, 0.0444; Taiwan, 0.0531; Thailand, 0.2621	2012	IMI (cases): 155	Overall (all-cause 90-day mortality): 32.9%	Multicenter, retrospective analysis of patient records	–
India^27^	IFD incidence (despite prophylactic treatment): 43.5%	2014–2016	AML (patients): 46. IFD patients: 20 (receiving posaconazole [*n* = 19]; amphotericin B [*n* = 1]; voriconazole [*n* = 0])	NR	Retrospective, single-center study	IFDs included cases of proven and probable infection while receiving induction chemotherapy and prophylactic antifungal therapy
Australia^28^	IFD incidence: 2.04% in hematologic malignancies; 6.29% in HCT recipients. Mould diseases incidence: 61% of all IFDs. IA incidence: most common mould identified, 5.1% in alloHCT recipients; 4.4% in AML patients	2005–2016	Hematologic malignancies (patients): 32 815. HCT recipients (patients): 1765. IFD (patients): 881. IA (patients): 31 alloHCT recipients; 29 patients with AML	NR	Observational, retrospective, longitudinal study of patients hospitalized in the state of Victoria and included in registries and databases	Development of IFD within 12 months of hospitalization increased risk of mortality by 1.24 in patients with hematologic malignancy

alloHCT, allogeneic hematopoietic stem cell transplantation; AML, acute myeloid leukemia; CLL, chronic lymphocytic leukemia; CPA, chronic pulmonary aspergillosis; CNPA, chronic necrotizing pulmonary aspergillosis; CS, corticosteroid; HCT, hematopoietic stem cell transplantation; IA, invasive aspergillosis; IC, invasive candidiasis; ICU, intensive care unit; IFD, invasive fungal disease; IMD, invasive mould disease; IMI, invasive mould infection; IPA, invasive pulmonary aspergillosis; MDS, myelodysplastic syndrome; NHS, National Health System; NR, not reported; PIMDA, prospective invasive mould disease audit.

In Middle Eastern and North African countries, invasive candidiasis, IA, chronic pulmonary aspergillosis (CPA), and mucormycosis feature among the IFDs reported in epidemiological studies, with IA and mucormycosis ranging in incidence from 0.6–16.7 to 0.034–9.2 per 100, 000 inhabitants per year, respectively.^15–19^ However, figures are likely to be inaccurate as registries of IFD are not available in these countries.

Data on IFD in the United States show varying incidence among HCT recipients depending on the source of the donor cells, ranging from 5.8 to 8.1 per 100 allogeneic HCT performed, while the incidence of IFD has been estimated to increase year-on-year by 0.24 cases per 100, 000 patients.^20,21^ In Brazil, the global incidence of IFD among patients with hematologic malignancies, including those undergoing HCT, has been reported as 9.6%–13.0%, with aspergillosis being the most common infection.^22,23^ While there is a lack of accurate published data on IFD incidence in Japan, a substantial temporal increase in the frequency of visceral mycoses was reported in autopsy cases over a 24-year period, from 4.5% in 1989 to 5.1% in 2013,^24^ and the incidence of IFD among patients with chronic lymphocytic leukemia (CLL) in the country has been reported as 3.9%.^127^ In another study from Japan, 55% of patients with clinical isolates of *Aspergillus* spp. collected from respiratory samples between 1998 and 2009 had a form of pulmonary aspergillosis, including 26.6% with chronic necrotizing pulmonary aspergillosis and 7.2% with invasive pulmonary aspergillosis.^25^ In other Asian countries, invasive mould infection incidence ranged from 0.0242 per 1000 patient days in China to 0.2621 per 1000 patient days in Thailand, with *Aspergillus* spp. as the most commonly cultured mould (71.6% of positive cultures).^26^ In a single center in India, 43.5% of patients with AML developed IFD while receiving induction chemotherapy, despite the use of prophylactic antifungal therapies.^27^ Few epidemiologic data have been published for Australia, but a study of hospitalized patients from 2005 to 2016 gave an incidence of IFD at 2.04% in patients with hematologic malignancies and 6.29% in those undergoing HCT, with mould diseases accounting for 61% of IFDs.^28^

### Pathogenic species

Since the focus of this review is isavuconazole, emphasis here is given to IFD involving *Aspergillus* spp. and the Mucorales, although it is important to acknowledge that the drug is active against other fungi, most notably, *Candida* spp. as well. *Aspergillus* spp., particularly those belonging to the *Aspergillus fumigatus* species complex, are the chief cause of invasive mould disease in most geographical areas.^20,29,30^ In Germany, these fungi are the most common mould infections, responsible for 1000–5000 IFDs annually and commonly occurring in patients with cell-mediated immune defects.^31^ In a 2-year retrospective survey of aspergillosis cases in Kuwait, *Aspergillus niger* complex was the most common isolate, involving 45% of cases, followed by *A. fumigatus, A. flavus, A. terreus*, and *A. nidulans* complex.^32^ Data from the TRANSNET surveillance study in the United States show that the cumulative incidence of *Candida* spp. infections among allogeneic HCT recipients was stable from 2001 to 2006, while during the same period there was a rise in the cumulative incidence of IA, from 0.6% in January–April 2003 to 2.8% in May–August 2004, with *A. fumigatus* dominating among the aspergillosis infections and *Nakaseomyces glabratus* (previously *Candida glabrata*) dominating among the invasive candidiasis infections.^20^ From 2004 to 2007, data from the Prospective Antifungal Therapy (PATH) Alliance registry revealed that among 234 adult HCT recipients, IA was the most common IFD (59.2%), followed by invasive candidiasis (24.8%), mucormycosis (7.2%), and other moulds (6.8%), with stable, temporal incidence of IA and IFDs caused by mucormycetes and other moulds contrasting with a decrease in the incidence of invasive candidiasis over the observation period.^30^

According to an epidemiological study of visceral mycoses from 1989 to 2015 in a national autopsy database of patients with hematologic malignancies and those undergoing HCT in Japan, *Aspergillus* spp. were the predominant causative agents, with decreasing prevalence of *Candida* spp. and increasing proportion of severe infections caused by Mucorales.^33^ The prevalence of *Aspergillus* spp. appeared to peak in 2005 but remained high thereafter. The incidence of mucormycosis is increasing, although its precise incidence is unknown because only a few population-based studies have been conducted and these studies differ in the populations enrolled and diagnostic procedures used.^34^ However, Mucorales are the next most common mould pathogen after *Aspergillus* spp. and have been known to be present as co-infections with *Aspergillus* spp. in up to 25% of published cases.^34–37^ In fact, one study using a Mucorales-specific polymerase chain reaction (PCR) assay in serum samples from patients with hematologic disorders (including those receiving intensive chemotherapy for acute leukemia or high-risk MDS and HCT recipients) at risk of IA, found that co-infection with *Aspergillus* was more common than mono-infection.^35^ While the exact burden of mucormycosis is not known, approximately 27 different species within the Mucorales order are known to cause infections, of which *Rhizopus arrhizus* is the most common agent, but other species within the *Rhizopus, Lichtheimia*, and *Mucor* genera are also implicated in disease.^38^ Emerging species implicated in mucormycoses are *Rhizopus homothallicus, Thamnostylum lucknowense*, and *Mucor irregularis*, among others.^38^

## Risk factors for fungal infections

The number and variety of patients at risk of IFD have expanded in recent years, owing in part to medical treatment advances. For instance, the number of HCTs performed in Europe almost doubled between 2000 and 2016, coinciding with the emergence of new at-risk populations, including hospitalized patients with severe influenza, a broader spectrum of hematological malignancies, chronic obstructive pulmonary disease (COPD),^31,39^ and severe acute respiratory syndrome-coronavirus-2 (SARS-CoV-2 [COVID-19]) infection.^40^ As alternative donor sources (umbilical cord blood, matched unrelated, or mismatched unrelated donors) have increased availability of HCT, so has the risk of developing IFDs.^39^

In addition to the above, other key risk factors for IFD include congenital immunodeficiencies such as chronic granulomatous disease and MonoMAC (monocytopenia and mycobacterial infection) syndrome, as well as immunosuppressant medications, including corticosteroids, used to prevent and treat transplant rejection following solid organ transplantation (SOT) and HCT.^4,31^ SOT recipients are at risk of IFD because of organ damage, neutropenia, and surgical factors, such as prolonged operation time and bleeding complications.^41^ In liver transplant recipients, independent risk factors for IA have been identified as previous fungal colonization, reoperation and previous bacterial infections, while after transplantation, renal replacement therapy, reoperation, and cytomegalovirus infections are known risk factors.^42^ Furthermore, tumor necrosis factor (TNF)-a inhibitors used for the treatment of autoimmune conditions (such as rheumatoid arthritis and inflammatory bowel disease) also modulate the immune response to fungal pathogens and increase the risk of IFD.^41^ To date, there are also numerous monoclonal antibodies (mAB) therapies (e.g., golimumab [anti-TNF-a], ofatumumab [anti-cluster of differentiation 20 (CD20)], infliximab [anti-TNF-a], and tocilizumab [anti-interleukin-6 (IL-6)]) that have been approved for the treatment of autoimmune diseases; these result in immunomodulatory effects that increase the susceptibility of the host to IFDs.^43^

Underlying lymphoproliferative disorders, such as CLL and non-Hodgkin’s lymphoma, also pose a higher risk of IFD.^44^ Previously, these patients have been reported to be at lower risk of infection with and mortality from, invasive mould infections than patients with blood disorders, such as AML, high-risk MDS, and acute lymphoblastic leukemia (ALL).^45,46^ However, the trend for increasing incidence of IFD in the former is thought to be linked to the use of more intensive treatment in these patients,^39^ which includes newer treatments (e.g., ibrutinib and venetoclax).^46^ Investigations of IFD in patients with hematologic malignancies, such as relapsed or refractory B-cell malignancies and multiple myeloma receiving chimeric antigen receptor (CAR)-T-cell therapy, suggest no increased risk of IFD owing to the relatively short duration of neutropenia (<500 cells/μl for ≤7 days).^47,48^ Yet, recent epidemiological studies have shown that IFDs still occur in approximately 2%–13% of patients who receive CAR T-cell therapy.^49^

Viral infections, such as COVID-19, also increase the risk of IFD.^50^ In a recent systematic review and meta-analysis from five medical databases, 3561 articles were identified following data searches; of these, 27 unique articles were included in the review (published between December 1, 2019, and July 27, 2023) following screening, with a total sample size of 6848 patients. Overall, 19.3% of patients with COVID-19 were also diagnosed with COVID-19-associated pulmonary aspergillosis (CAPA), and diagnosis rates of CAPA ranged from 2.5% to 47.2%.^50^ Eight risk factors for CAPA were identified, including pre-existing comorbidities of chronic liver disease, hematological malignancies, COPD, and cerebrovascular disease.^50^ Additionally, use of invasive mechanical ventilation, use of renal replacement therapy, treatment of COVID-19 with an interleukin-6 inhibitor, and treatment of COVID-19 with corticosteroids were shown to be associated with CAPA.^50^ Notably, in contrast to patients without CAPA, those with CAPA were also typically older (mean age: 66.6 years vs. 63.5 years), had a longer duration of mechanical ventilation (mean duration: 19.3 days vs. 13.5 days), and had higher all-cause mortality (odds ratio [OR]: 2.65).^50^ Subsequently, an increase in cases of COVID-19-associated mucormycosis associated with high mortality and morbidity was reported predominantly in India,^51^ with an incidence of 0.14 per 1000 people.^52^ In contrast, lower occurrences of mucormycosis were observed in Europe.^53^ In a study of 1035 high-risk critically ill COVID-19 patients in the Netherlands, all cultures were negative for Mucorales, whereas 42 (11%) cultures were positive for *Aspergillus*.^53^

Less well-defined subgroups of patients, such as those requiring admittance to an intensive care unit (ICU), may also be at increased risk of IFD.^31^ Risk factors for IA in non-neutropenic patients in the ICU include prolonged corticosteroid treatment prior to ICU admittance and prolonged (>7-day) ICU stay.^54^ Patients in the ICU undergo a variety of therapies and procedures, such as broad-spectrum antibiotics, mechanical ventilation, and insertion of a central venous catheter, which may impact the immune defense system and, alongside contributing factors linked to critical illness, could result in invasive IFD.^54^ Liver cirrhosis has also been linked to increased incidence of IFD, with invasive candidiasis and IA being the two most common.^54,55^ There is increasing recognition that IFDs are underdiagnosed and associated with high morbidity and mortality in individuals with acute or chronic liver impairment, with invasive pulmonary aspergillosis in particular causing high mortality rates in patients with severe alcoholic hepatitis.^56^ COPD, particularly for more advanced stages of the disease (GOLD stage III–IV), is associated with increased incidence of IA, likely because of corticosteroid prescribing practices, impaired immunologic response alongside reduced mucociliary clearance, and exposure to *Aspergillus* spp.^54,57^

Lastly, mucormycosis is increasingly reported in patients with uncontrolled diabetes mellitus (particularly in Asia), those undergoing corticosteroid therapy, and those with hematologic malignancy and solid organ transplantation, particularly in Europe and the United States.^38^

## Diagnosis of invasive fungal infections

Generally, diagnosis of IFD is based on clinical examination, imaging, and confirmation of the presence of the causative agent,^31,41^ and it is increasingly recognized that risk stratification of patients based on underlying conditions, procedures, and treatments may aid in the prompt diagnosis and treatment of IFD.^58^ Subsequently, sufficient access to suitable diagnostic tools is also a crucial factor in achieving an early diagnosis of IFDs.^59,60^

Culture-based diagnostic techniques are considered the gold standard for identification of pathogenic fungi^61,31^ and, together with antifungal susceptibility testing, they are enhanced by non-culture-based assays.^62^ However, culture is hampered by long turn-around time and low sensitivity.^63,64^ In addition, culture may also have differing yields for fungal pathogens depending on specimen type. For instance, the moulds are rarely isolated from cerebrospinal fluid or blood cultures, whereas *Aspergillus* spp. are readily cultured from bronchoalveolar lavage specimens.^63^

Direct microscopic imaging examining the morphological features of a fungal pathogen in biopsy tissue or fluid does not rely on fungal culture and enables differentiation based on histopathology, but it is not sufficient alone to identify a pathogen to the species level.^63^ Direct histopathological examination of tissue, such as skin biopsy for *Fusarium* spp., could give rise to rapid results before culture findings are available.^61^ Furthermore, direct microscopic imaging and histopathologic analysis may be useful to avoid false negative results from fungal culture.^63^ Non-culture based assays include mannan/anti-mannan immunoassay, 1,3-β-d-glucan (BDG) testing, T2 magnetic resonance (T2MR) and PCR assay for candidiasis, and galactomannan immunoassay (including lateral flow assays), BDG testing, and PCR assay for aspergillosis; of which, PCR and T2MR (candida only) offer the fastest results with good sensitivity and specificity at the species level.^64^

While a variety of diagnostic techniques are available for IFD, their geographic accessibility differs widely. In European countries, for instance, quantitative *Aspergillus* spp. PCR and BDG testing are not widely available, while access to galactomannan antigen testing varies according to the type of specimen (serum/blood or bronchoalveolar lavage).^65^

Access to culture media and microscopy was available in ≥97% of sites in 45 European countries surveyed by the European Confederation of Medical Mycology, but there was wide variation in the availability of molecular-based tests, such as PCR.^60^ In a survey of centers in 40 countries or territories in the Asia/Pacific region, including India, China, Thailand, Indonesia, Iran, Australia, and Japan, antigen detection testing was available in 79% of sites, access to PCR and other molecular tests was reported at 66% of sites, and antibody detection tests were available in only 63% of sites.^66^ In the United States and Canada, suboptimal diagnostic approaches for the detection of yeast and mould from blood cultures derived from patients suspected of having IFD and a lack of a molecular detection assay for mucormycosis were two gaps identified in the laboratory diagnosis of fungal diseases in the region.^67^ While identified as being useful for polymicrobial fungal infections, next-generation sequencing is a newer technology that has been explored to identify fungi in formalin-fixed paraffin-embedded tissue, although the technique is currently costly, time-consuming, and requires highly skilled and trained technologists.^67^ Diagnosis of mucormycosis is dependent on the availability of appropriate imaging techniques and mycological and histological investigations, which may include immunohistochemistry with commercially available mAB or PCR techniques,^68^ the availability of which may be dependent on the gross domestic product of a country.^60,66^

Matrix-assisted laser desorption ionization-time of flight mass spectrometry (MALDI-ToF MS) is another tool used for fungal identification and has been successfully employed to identify *Candida* spp., *Aspergillus* spp., and other moulds.^69^ The availability of MALDI-ToF MS has been continually increasing worldwide,^70^ and is now commonplace in most clinical microbiology laboratories, offering rapid, accurate, and highly reproducible results.^69^ Furthermore, MALDI-ToF MS has been extended to develop antifungal susceptibility tests for fungi such as *Candida* spp. and *Aspergillus* spp., providing a rapid method for determining the susceptibility of pathogens to antifungal drugs.^69^

Whether antifungal susceptibility testing is performed routinely varies from region to region. Currently, the European Society of Clinical Microbiology and Infectious Diseases (ESCMID) recommends that resistance testing of *Aspergillus* spp. isolates responsible for causing IFD is conducted in regions where azole resistance appears in contemporary surveillance programs.^31^

In relation to the diagnosis of aspergillosis and mucormycosis, PCR is not readily available in the United States or Latin America, BDG testing and the mannan/anti-mannan immunoassay are not widely available in Latin America;^71^ and BDG testing is not available in Australia.^72^ In Kuwait, both culture/non-culture-based assays and an in-house PCR assay are used for the detection of *Aspergillus* spp. infections; all clinically significant isolates undergo antifungal susceptibility testing and resistance gene sequencing.^16^ In general, *Candida* spp. are routinely tested for antifungal susceptibility, while susceptibility testing in other mycoses is less commonly performed. When an Aspergillus isolate is obtained, it is typically recommended that susceptibility testing be performed, although a majority of patients are still diagnosed by non-invasive methods, decreasing the availability of isolates for testing. Among the rare moulds, susceptibility testing is also commonly performed in an attempt to optimize antifungal therapy, but clinical outcomes have not been clearly linked to *in vitro* susceptibility;^73^ although treatment decisions in high-risk patients are often required before such data are available. This is evidenced by a retrospective study in patients with invasive fusariosis who demonstrated a lack of any correlation between mortality rates and minimum inhibitory concentration by 6 weeks after the diagnosis.^74^

A non-invasive liquid biopsy (Karius Test^®^, Redwood City, CA, USA) is used in the United States to detect cell-free DNA of pathogens, such as *Aspergillus* spp. In Japan, antifungal susceptibility testing of aspergillosis isolates is generally only performed by reference laboratories, and whole-genome sequencing (WGS) is conducted for research rather than clinical purposes. In contrast, in the United States, WGS is used only for investigation of outbreaks, and in Kuwait, it is used for cases of difficult-to-treat, pan-drug-resistant isolates.

In Australia, access to laboratory tests is generally good; each jurisdiction (state or territory) has its own reference laboratory, but in two of the largest states, New South Wales and Victoria, testing is decentralized. Most hospitals have ready access to culture-based methods and basic molecular methods, e.g., *Aspergillus* PCR and pan-fungal PCR; WGS is generally undertaken for research purposes. Antifungal susceptibility testing for yeasts is available widely and for moulds at most reference labs.

## Clinical use of isavuconazole

Isavuconazonium sulfate (active moiety: isavuconazole) is an advanced generation triazole antifungal and has demonstrated activity against a variety of yeasts, moulds, and dimorphic fungi, both *in vitro* and in animal models.^75^ It can be administered orally or intravenously, and is approved for the treatment of IA and mucormycosis based on pivotal phase 3 clinical trials.^6^ The SECURE double-blind, randomized, comparative (vs voriconazole) study and VITAL single-arm, open-label study were phase 3, multicenter clinical trials demonstrating the efficacy and safety of isavuconazole for the treatment of adults with IFD caused by *Aspergillus* spp. or other filamentous fungi, including mucormycosis. Furthermore, in the SECURE study, isavuconazole was shown to be non-inferior to voriconazole in terms of all-cause mortality while being associated with significantly fewer drug-related adverse events.^76,77^

### Real-world effectiveness and safety of isavuconazole

While treatment decisions in clinical practice are based on guideline recommendations, real-world evidence can provide a broader perspective based on the often complex scenarios of IFD seen in practice, where the presence of drug–drug interactions and severe drug-related adverse events, for instance, preclude the use of a guideline-recommended antifungal.^78^ Real-life patient populations that receive treatment may also be substantially different from patients selected for randomized clinical trials (RCTs), as RCTs only include patients who meet specific eligibility criteria; subsequently, patients who are severely ill or with refractory underlying conditions or organ failures are unlikely to be enrolled in RCTs.

In a small study of patients with leukemia (*n* = 23) and evidence of azole-induced hepatotoxicity or grade 3–4 QTc prolongation while on posaconazole, a switch to isavuconazole was well tolerated with no discontinuations due to toxicity, with reduced liver function test values and resolution of QTc abnormalities.^79^ In solid organ transplant recipients, interactions with calcineurin and mTOR inhibitors and adverse drug reactions may limit the use of triazole antifungals other than isavuconazole. Additionally, in a non-comparative, observational study of 53 patients with severe comorbid conditions and IFD (mainly due to *Aspergillus* spp.), isavuconazole was well tolerated and effective (clinical cure at end of treatment 50.9%).^80^

In an observational, retrospective study of 122 patients with hematologic malignancies, isavuconazole, used as first-line therapy (35%) or subsequent-line therapy (65%), resulted in a radiologic response rate of 67.2% (with respective complete and partial radiologic response rates of 51% and 47%) and a high radiologic response rate of 81.6% in those with IFD refractory to prior antifungal treatment.^81^ Evidence from a retrospective, multicenter, international real-world study of isavuconazole, voriconazole, and amphotericin B in 112 patients at high risk of IFD revealed no difference between the treatments in terms of response to primary therapy or mortality.^82^ In this study, the majority (79%) of identified organisms were *Aspergillus* spp., followed by *Fusarium* spp. (8%), *Mucor* spp. (6%), *Trichosporon* spp. (3%), and others (4%);^82^ a favorable response to isavuconazole therapy was recorded in 90% of patients.^82^

In an observational, multicenter case series study from China, 40 patients with a range of comorbidities (including hematologic malignancies, sepsis, pulmonary mycosis, graft-versus-host disease [GVHD], and allogeneic HCT) received isavuconazole for the treatment of IFD (primarily IA and IM).^83^ Clinical response was achieved in 75% (30/40) of these patients; response rates were 66.7% (8/12) for those with IA, 83.3% (10/12) for those with IM, and 0% (0/2) for those with invasive candidiasis, with 10% (4/40) of patients reporting isavuconazole-related adverse events and no discontinuations due to adverse events.^83^

A study of 82 patients with coccidioidomycosis, including some with pulmonary (38%), bone/joint (13%), and central nervous system (41%) involvement, and prior antifungal treatment (including amphotericin B, fluconazole, itraconazole, voriconazole, and posaconazole) found that isavuconazole was associated with improved outcomes (reflecting changes in clinical findings and Mycosis Study Group [MSG] score) in 70% of patients, no change in 21% of patients, and worsening condition in 10% of patients.^84^ Three patients discontinued due to possible adverse events (palpitations, transaminitis, and hot flashes), although none had any worsening in MSG score.^84^ In a smaller study of patients with coccidioidal meningitis (*n* = 9) who received initial treatment with fluconazole and second-line treatment with posaconazole or voriconazole before transitioning to isavuconazole, assessment at a mean isavuconazole treatment duration of 504 days revealed treatment success (by MSG scoring criteria in three patients and stable disease in six patients, with clinician-assessed treatment success in five patients and stable disease in four patients) and no treatment failures.^85^ During the observation period, no treatment failures related to isavuconazole were identified, although one patient discontinued treatment due to worsening of pre-existing dyspepsia.^85^

In a meta-analysis of isavuconazole studies for the treatment of IFD, mortality as evaluated in six studies (870 patients) was not significantly inferior to that with other antifungals (OR 1.11, 95% confidence interval [CI] 0.82–1.51; *I*^2^ = 0), with numerically lower mortality with isavuconazole than control antifungal therapy (28.3% vs 33.6%, respectively). The discontinuation rate with isavuconazole was significantly lower than that for control antifungal (9.8% vs 16.9%, respectively);^86^ the incidence of hepatic function abnormalities was also significantly lower with isavuconazole than for control antifungal (8.0% vs 16.3%; OR 2.31).^86^

In clinical trials, isavuconazole was shown to be well tolerated with a favorable safety profile compared to other azole antifungals;^87^ the most common adverse events reported in trials were nausea, vomiting, and diarrhea, with very few patients requiring treatment discontinuation.^87^ In the SECURE phase 3 comparative trial, permanent drug discontinuations due to treatment-emergent adverse events (14% vs 23%, respectively) and drug-related adverse events (8% vs 14%, respectively) were less common with isavuconazole than with voriconazole.^76^ However, because of the potential for liver toxicity, liver function monitoring is advised.^6^

In a retrospective, multicenter, international real-world study of IFD treatments, isavuconazole was associated with significantly fewer adverse events than voriconazole and amphotericin B.^82^ In particular, isavuconazole was superior in terms of liver toxicity compared to voriconazole, with lower rates of renal failure compared to amphotericin B-based regimens.^82^ Furthermore, in a retrospective real-world study in adults with hematologic malignancies, use of isavuconazole still demonstrated a promising clinical response and a favorable safety profile, including patients that had previously failed to respond to other azole therapies (i.e., voriconazole, posaconazole).^88^ Additionally, a single-center, retrospective study in lung transplant recipients showed similar efficacy for both isavuconazole and voriconazole as antifungal prophylaxis, with fewer adverse events linked to early discontinuation occurred for isavuconazole (11% vs 36%).^75^

A particular concern in relation to the use of triazole antifungals is the interaction with targeted chemotherapies used in the hematologic malignancy setting, such as ruxolitinib, and venetoclax, which undergo extensive hepatic metabolism.^82^ However, isavuconazole has a lower propensity for interaction with these therapies than voriconazole and amphotericin B.^82^ Notably, patients with hematologic malignancies receiving isavuconazole in the real-world setting have demonstrated similar outcomes for both monotherapy and combination therapy (i.e., polyene, echinocandins, or terbinafine).^88^

Unlike other triazole antifungals, isavuconazole does not prolong the QTc interval and in fact shortens the QTc interval by 5 msec, which appears to be an advantage when treating IFD in patients who often require concurrent treatment with therapies that prolong the QTc interval or have comorbidities that have this effect. However, isavuconazole is contraindicated in patients with familial QT syndrome.^6^ There is currently a lack of data on the combination of isavuconazole and amiodarone, and therefore uncertainty about a potential drug–drug interaction.^75^

Isavuconazole showed good *in vitro* activity against 208 clinical and environmental *Aspergillus flavus* isolates from India and The Netherlands, with minimum inhibitory concentrations of ≤2 μg/ml in 98.9% of isolates.^89^

### Prophylactic use of isavuconazole

According to guidelines, mould-active prophylaxis is recommended in those with prolonged neutropenia resulting from chemotherapy for AML or MDS and in recipients of HCT requiring augmented immunosuppression for GVHD.^68,75,90^ Recent recommendations of the AGIHO/DGHO state that isavuconazole might be considered as primary or secondary antifungal prophylaxis in long-term neutropenic hematology patients.^47^ However, other countries lack specific guidelines for prophylactic use of isavuconazole.

While isavuconazole is not licensed as a prophylactic treatment in patients at high risk of IFD, there is nonetheless growing evidence for its use as a mould-active prophylaxis owing to its favorable tolerability and pharmacokinetic profiles, low propensity for drug–drug interactions, and lack of QTc interval prolongation.^75^ Indeed, published data, including a phase II open-label prospective study in patients with AML or MDS, were reviewed extensively in Lewis et al., and while a head-to-head comparison of isavuconazole and other triazole antifungals in a prospective, randomized setting has not been done, the authors concluded that evidence to date suggest largely comparable efficacy.^75^

More recently, patients who received primary prophylaxis with isavuconazole during AML induction therapy or post-HCT experienced a similar incidence of IFD compared with those administered posaconazole, according to two single-center retrospective studies from the United States^91,92^ with the authors suggesting that the choice of prophylactic antifungal should be guided by patient factors such as concomitant medications and baseline QTc interval.^91^ A recent retrospective, matched cohort study conducted in patients with AML, high-risk MDS, and those who had undergone HCT at a single center in the United States found a numerically higher incidence of breakthrough IFD in the isavuconazole group (16.7%) than in the posaconazole and voriconazole groups (10.7%), although differences were not statistically significant (*P* = .67), and hepatotoxicity was more common among posaconazole recipients (17.3%) than in isavuconazole recipients (4.8%).^92^ In a further retrospective, single-center cohort study from the United States in which 106 patients with a history of hematologic cancer or cellular therapies (allogeneic or autologous HCT, or chimeric antigen receptor T-cell therapy [CAR-T]) received isavuconazole for ≥7 days as primary or secondary prophylaxis, there was a cumulative incidence of 17.9% breakthrough IFD (12.3% were proven or probable), with these occurring in patients with a relatively long median duration of isavuconazole (78 days).^93^ A large subgroup analysis of antifungal prophylaxis in high-risk patients (*n* = 1177) included in a multicenter, observational, prospective registry in the United States found that breakthrough IFD in those with assessment results were similar with isavuconazole (5.0%), posaconazole (5.3%), and voriconazole (4.0%), and that the proportion of discontinuations due to adverse drug reactions was numerically lower among isavuconazole recipients (2.0%) than posaconazole (8.2%) and voriconazole (10.1%) recipients.^94^

The review by Lewis et al.^75^ also included a retrospective study of isavuconazole and voriconazole prophylaxis in patients who had undergone lung transplantation, with a similar incidence of breakthrough IFD in both groups (3.5% and 3.2%, respectively), although isavuconazole had superior tolerability to voriconazole, with a significantly lower incidence of premature discontinuation due to adverse events (11% vs 36%, respectively; *P* = .0001).^95^

A recent pooled analysis of isavuconazole for prophylaxis against IFD revealed no significant difference in the incidence of IFD with isavuconazole and control antifungals (OR 1.02, 95% CI 0.49–2.12; *I*^2^ = 0%) in 577 patients analyzed, with significantly lower incidence of hepatic function abnormalities (3.6% vs 11.9%, respectively; OR 3.63).^86^

### Isavuconazole in specific populations

There are many challenges in the management of IFD in the setting of underlying malignancies and transplant populations, including liver toxicity, drug interactions, renal dysfunction, diabetes mellitus, older age, persistent neutropenia or lymphopenia, and prolonged QTc interval.^82^ Patients with rare IFDs may also be candidates for isavuconazole treatment. A wide range of pediatric patients are at risk of IFD and could potentially be treated with isavuconazole.

*Histoplasma capsulatum* causes high mortality in individuals with advanced HIV infection. Liposomal amphotericin B and itraconazole are the preferred treatments, but due to concerns with organ failures, toxicity, drug interaction, and therapeutic plasma levels, they may be difficult to use. In a reported case of disseminated histoplasmosis, treatment with itraconazole and posaconazole failed to attain therapeutic levels.^96,97^ Following a switch to long-term isavuconazole, the patient experienced resolution of symptoms and complete clinical recovery at 1-year follow-up.^98^ Isavuconazole was also used to successfully treat disseminated histoplasmosis in a patient with rheumatoid arthritis receiving methotrexate and infliximab, and who was unable to be treated using itraconazole or amphotericin B.^99^ A case of upper extremity *H. capsulatum* infection was also successfully treated with 3 months of isavuconazole therapy.^100^

In patients with and without hematologic malignancies, *Trichosporon* spp. are a common cause of breakthrough fungemia, particularly following exposure to echinocandins, given their inherent resistance, that pose a substantial mortality risk.^101^

Recent *in vitro* data suggest that isavuconazole may play a role in this patient setting, as variable *in vitro* activity has been reported against clinically relevant *Trichosporon* spp. isolates from Brazil, although there was evidence of potential triazole cross-resistance in some *Trichosporon asahii* non-wild-type isolates.^102^ Furthermore, in two patients with hematologic malignancies and *T. asahii* infections, isavuconazole provided clinical success in both patients by the end of treatment, despite one having an infection refractory to prior antifungal treatment.^103^ Similarly, a patient with ALL and *T. asahii* fungemia who ceased voriconazole treatment because of neurological toxicity was subsequently successfully treated with isavuconazole.^104^

Isavuconazole has also been suggested as an alternative antifungal option for patients with AML on IDH1/2 inhibitors, such as ivosidenib, as the other azole antifungals and IDH1/2 inhibitors prolong the QTc interval, while isavuconazole has no QTc prolongation effects.^105^

Currently, the intravenous use of voriconazole, itraconazole, and posaconazole requires coadministration with sulphobutylether-β-cyclodextrin, which may accumulate in those with impaired renal function and is associated with renal dysfunction when administered with other drugs such as penicillins, fluoroquinolones, and immunosuppressants.^106^ Patients with renal impairment may be offered intravenous isavuconazole rather than other azole antifungals owing to the lack of cyclodextrin as an excipient.^80^ In other indications, isavuconazole’s similar spectrum of antifungal activity makes it a valid alternative to posaconazole for primary prophylaxis against invasive mould infections in HCT and GVHD, but data on whether isavuconazole and posaconazole have equivalent effectiveness in the setting of HCT recipients with acute GVHD is limited.^75^ In a retrospective, single-institution study of adult patients with hematologic malignancies who were HCT recipients and received ≥7 days of isavuconazole primary prophylaxis, an increased rate of breakthrough IFDs, in particular invasive pulmonary aspergillosis (6.8%), was reported with isavuconazole compared with both posocanazole (1.3%) and voriconazole (0%), although this comparison did not reach statistical significance.^107^

Additionally, although supporting data are limited, the considered opinion from this author group was that the availability isavuconazole as of both oral and intravenous formulations provides an advantage for the treatment of IFDs over antifungals with only a single route of administration.

However, isavuconazole should be avoided in patients taking potent CYP3A4 inducers, such as rifampicin, phenytoin, carbamazepine, phenobarbital and ritonavir, owing to reduced isavuconazole exposure.^108^ Similarly, isavuconazole should be avoided in patients taking strong CYP3A4/5 inducers such as aprepitant, prednisone, and pioglitazone.^6^ Caution is also advised when administered with strong CYP3A4/5 inhibitors such as lopinavir/ritonavir, while co-administration with ketoconazole is contraindicated.^6^ Furthermore, caution is advised when administering isavuconazole with agents affected by P-gp efflux.^6^ Isavuconazole should also be avoided in patients with severe hepatic impairment, as isavuconazole has not been studied in these populations.^6^ Treatment with isavuconazole is generally not recommended during pregnancy due to potential risks to the unborn baby, except in patients with severe potentially life-threatening fungal infections where the anticipated benefit would outweigh the risks.^6^ In a case report involving treatment of Aspergillus lung infection in late pregnancy with multiple anti-fungal drugs, treatment with isavuconazole resulted in the resolution of infection, ultimately leading to the delivery of a healthy newborn at term.^109^

Isavuconazole has only recently been approved by both the FDA and EMA for the treatment of IA and IM in pediatric patients.^6,7^ However, isavuconazole use in pediatric clinical practice has been documented prior to receiving regulatory approval. Although real-world use of isavuconazole in children with IFD (including immunocompromised patients) is limited to small retrospective studies and case reports, findings suggest isavuconazole was effective and well tolerated at similar doses to those used in the adult regimen.^110–114^

### Therapeutic drug monitoring in a real-world setting

Evidence suggests that therapeutic drug monitoring (TDM) of isavuconazole may be warranted in patients who are obese, <18 years of age, or who have moderate hepatic failure.^87^ There was also a suggestion that subtherapeutic levels of isavuconazole could lead to higher rates of clinical failure in obese patients than in non-obese patients in a clinical trial of invasive candidiasis.^108,115^ A further real-world retrospective observational study conducted on isavuconazole use (5.4 mg/kg up to 200 mg) in children from 2018 to 2021 demonstrated that a high proportion of patients, particularly those with ≤35 kg body weight, had trough concentrations outside of the therapeutic range; as such, pediatric patients could benefit from early and systematic TDM.^116^

As isavuconazole is a moderate inhibitor of CYP3A4/5, it is advised that TDM is conducted during co-administration with medicines metabolized by CYP3A4, including the immunosuppressants tacrolimus, sirolimus, mycophenolic acid, and cyclosporine.^6,108^ However, current guidelines do not definitively recommend the need for routine TDM, but instead state that it could be useful for the clinical assessment or monitoring of patients receiving isavuconazole who do not respond to treatment, develop unexpected toxicity or drug–drug interactions, or if isavuconazole is used to treat pathogens with elevated minimum inhibitory concentrations or infections at sites such as the central nervous system.^90^

With few published data on TDM of isavuconazole serum levels in clinical practice, Kosmidis et al. studied its role during long-term oral isavuconazole treatment (range 18–1473 days) in 45 patients with CPA.^117^ The authors found that adverse events were more likely in patients with an isavuconazole threshold above 4.6 mg/liter, but that the administered daily dose, rather than drug level, was predictive of serious adverse events.^117^ In line with this, the authors observed evidence of toxicity at widely varying drug blood levels, both above and below this threshold. Limited data are available for patients receiving renal replacement therapy or extracorporeal membrane oxygenation.^108^

In general, studies are still conflicted regarding the necessity of TDM for isavuconazole and its impact on clinical outcomes and reduction of toxicity in a real-world setting;^75,87^ however, several key data have been reported. Serum concentrations were shown to have a lower degree of variability in patients receiving isavuconazole versus voriconazole in a Danish institute (Statens Serum Institute).^118^ Additionally, in a retrospective study of Indian patients, 10% had subtherapeutic serum levels of isavuconazole (using a cutoff of 2 mg/l) serum exposure following oral administration.^119^ Lastly, subtherapeutic levels of isavuconazole have also been reported when administered in combination with flucloxacillin despite standard dosing, potentially necessitating the use of TDM to ensure an adequate exposure.^120,121^

### Resistance to isavuconazole

Resistance to isavuconazole varies widely between regions but has not generally been encountered in clinical practice. However, there have been reports of triazole treatment failure in many countries, including India, China, Iran, Tanzania, Australia, the United States, and European countries, because of triazole-resistant *A. fumigatus* induced by *CYP51A* gene mutations, such as TR34, L98H, and TR46 Y121FT289A, which may be linked to the agricultural use of triazole antifungals.^122^ The *in vitro* minimum inhibitory concentration of isavuconazole was reported to be very high (>16 μg/ml) against isolates harboring these mutations.^122^ Data on the antifungal susceptibility of *Aspergillus* spp. isolates from the Arab League countries are scarce, although it is thought that voriconazole resistance in *A. fumigatus* and other *Aspergillus* spp. is not increasing in the region to the same extent as in other geographical regions.^15^ This is noteworthy since other studies of isolates from various geographical regions suggest that isavuconazole *in vitro* potency is similar to that of voriconazole.^75^ However, data on isavuconazole resistance among pathogenic *Aspergillus* spp. isolates from Japan are not yet available.

*Candida albicans* and *N. glabratus* (previously *C. glabrata)* have different azole susceptibility profiles and known azole resistance mechanisms. Susceptibility testing of their clinical isolates revealed that resistance mechanisms involving ATP-binding cassette (ABC) transporters and lanosterol 14-α-sterol-demethylase (*ERG11*) decreased the activity of isavuconazole, while mechanisms involving mutations in the major facilitator (*MDR1*) allele had little effect.^123^

Isavuconazole demonstrates good *in vitro* activity against some isolates of the order Mucorales responsible for mucormycosis, including *Lichtheimia, Rhizopus*, and *Rhizomucor* spp. Including *R. arrhizus* (*oryzae*), the most commonly cultured Mucorales member in patients with mucormycosis. Reduced isavuconazole *in vitro* susceptibility has been reported for *Mucor* spp.^75^ Both isavuconazole and posaconazole are believed to have species-specific activity within the Mucorales, which stresses the importance of accurate species identification.^75^

Reduced susceptibility to isavuconazole, itraconazole, voriconazole, fluconazole, and anidulafungin has also been reported for a clinical *Sporothrix schenckii* isolate causing sporotrichosis in one patient, with the strain speculated as having acquired a resistance mechanism rather than being innately non-susceptible.^124^

## Future perspectives

Treatments for acute leukemia and MDS, especially HCT, are associated with aggressive suppression of the immune system and the subsequent widespread use of antifungal prophylaxis, resulting in changes to the epidemiology of disease-causing fungi, with an increasing incidence of breakthrough infections and drug-resistant pathogens, such as non-*C. albicans* spp., azole-resistant *Aspergillus* spp., or Mucorales.^12,33^ Consequently, it is imperative for clinicians to focus on these infections in at-risk patients. Isavuconazole is an important option in the management of patients at risk of IFD, particularly when toxicities, pharmacokinetics, or drug interactions preclude the use of voriconazole or posaconazole.^75^ However, there is a need for novel agents with different modes of action, including the use of older agents with alternative routes of administration, with universal access in all geographic regions. The use of combination antifungal therapies may also be of interest for critically ill patients with IFD, owing to the potential for a reduced mortality rate.^125^ Alongside this, stewardship programs for specific scenarios are needed to direct appropriate use of antifungals to achieve the best clinical outcomes and minimize resistance development.

Currently, there is regional and global disparity in terms of access to essential fungal diagnostic testing, despite the critical need for rapid identification of IFD to ensure early treatment and prevent severe disease and death.^126^ Enhanced capacity of laboratories around the world is crucial to prevent disease and deaths caused by IFD, including the development of rapid and reliable diagnostic tools and expansion of training programs to develop expertise in fungal diagnostics.^126^ The incorporation of fungal pathogens into existing surveillance programs would be beneficial for tracking infections and the spread of resistant pathogens to guide public health activities, while establishing fungal registries in all countries would also be of benefit. These arguments are also supported by the recent fungal pathogen priority list published by the WHO,^2^ in an attempt to drive further research and strengthen the global response to fungal infections and antifungal resistance. However, although some global and regional guidelines exist for aspergillosis, mucormycosis, endemic mycoses, and rare mould infections, more country-specific guidelines are required to incentivize public and private health systems to fund necessary diagnostic tests and the antifungal treatments needed.
